# Declines in grip strength may indicate early changes in cognition in healthy middle-aged adults

**DOI:** 10.1371/journal.pone.0232021

**Published:** 2020-04-23

**Authors:** Diane E. Adamo, Tara Anderson, Mahtab Koochaki, Nora E. Fritz

**Affiliations:** 1 Program in Physical Therapy, Wayne State University, Detroit, Michigan, United States of America; 2 Institute of Gerontology, Wayne State University, Detroit, Michigan, United States of America; 3 Department of Neurology, Wayne State University, Detroit, Michigan, United States of America; Kessler Foundation, UNITED STATES

## Abstract

Declining grip strength is an indicator of cognitive loss in older individuals but it has not been explored people younger than 65 years old. The purpose of this study was to investigate the relationship between grip strength and specific cognitive tests known to decline with mild cognitive impairment in young and middle-aged adults. Declines in cognitive performance in middle-aged adults may provide evidence that these changes occur earlier than previously reported. A cross sectional design was used to compare differences between young and middle-aged healthy adults and to investigate associations between cognitive and grip strength measures within groups. Healthy young (20–30 years old) and middle-aged (45–65 years old) adults completed five cognitive tests including the Stroop, California Verbal Learning Test, Symbol Digit Modalities Test, Trail Making Tests and the Controlled Oral Word Association Test. All participants completed right and left maximum grip strength measures. Middle-aged adults performed significantly worse on right and left grip strength and the Stroop test (p<0.05) when compared to the younger group. There were no significant relationships among grip strength and cognitive performance at the whole-group level or within the younger-age group; however, weaker grip strength was significantly associated with poorer Controlled Oral Word Association Test total cluster (r = 0.458; p < .05) and Stroop interference (r = 0.471; p < .05) scores in the middle-aged group. Findings from this study suggest that cognitive changes may occur earlier than previously thought (prior to age 65). Weaker grip strength was significantly associated with poorer function in two of the cognitive measures in the middle-age group, suggesting that some domains of cognition, specifically semantic categorization and executive function, may be particularly sensitive to age-related changes.

## Introduction

The relationship between cognition and physical function has been well studied in older adult populations [1]. Higher physical function is associated with better cognitive performance in older cohorts [2]. Better cognitive performance is associated with faster walking speeds [3, 4, 5] and slower walking speeds are known to precede dementia syndromes in longitudinal studies [3]. In addition to walking speed, declining grip strength has been shown to be an early functional biomarker of cognitive decline for individuals with mild cognitive impairment (MCI) and dementia [2, 6, 7].

A handgrip dynamometer is an inexpensive and reliable instrument used to measure grip strength [8]. Grip strength can be easily measured in a clinical setting when other functional measures such as gait speed may be limited due to specific health restrictions (e.g. assistive devices) [5]. Reduced grip strength has also been shown to be a marker of overall physiological function in elderly adults [9] even in the presence of differences in physical condition, exercise habits, and history of injury. Decreased hand grip strength correlates with lower scores in several cognitive domains including executive function, attention, working memory, language and semantic categorization, and overall cognition in non-demented older adults [6,7].

Strong associations have been found between cognitive performance and grip strength scores, and these associations are particularly evident when cognitive domains require motor functions [9]. A study by Boyle et al. [10] found that elderly participants with a high level of muscle strength had a 48% decreased risk of developing MCI compared to participants with a low level of muscle strength. Interestingly, the majority of studies examining grip strength have focused on individuals aged 65 years and older [2, 7, 9, 11–14] since the steepest decline in cognitive ability occurs after age 65 [12]. However, the relationship between grip strength and cognition has not been explored in young and middle-aged adults, despite recent evidence that handgrip dynamometry has the potential to serve as a “vital sign” for brain health in middle-aged and older adults [15].

Cognitive decline is a longitudinal process; indeed, in MCI, early brain changes are present prior to overt clinical symptoms [16] supporting the progression of early cognitive changes, perhaps earlier than originally thought. Therefore, diagnostic biomarkers are needed to detect early cognitive changes that may be indicative of MCI or Alzheimer’s disease (AD). Examination of physical and cognitive performance in middle-aged individuals may provide evidence that these changes occur earlier than previously reported [17]. With declining cognitive capacity, the ability to monitor changes in motor and cognitive function is crucial to identifying specific interventions that may intercede and reduce potential loss. [5] Examination of both physical and cognitive functioning may help clinicians better identify individuals who age differently and at different rates [2].

The purpose of our study was to investigate the relationship between grip strength and cognitive function in young and middle-aged adults. We hypothesized that middle-aged adults would demonstrate lower cognitive performance and lower grip strength than younger adults and show stronger correlations between grip strength and specific cognitive domains known to decline with MCI, including executive function and semantic categorization/phonemic fluency. Thus, the results of this study may inform future work, including longitudinal designs to establish a predictive relationship between cognitive performance and grip strength in individuals under age 65.

## Methods

### Participants

A cross sectional within-subjects design included a convenience sample of 51 participants who were recruited from posted flyers at Wayne State University, the Rehabilitation Institute of Michigan, local grocery stores, libraries, and community centers. Participants were eligible to participate if they met the age criteria of 20–30 years for young and 45–65 years for middle-aged groups, could grip without difficulty, had normal or corrected vision, and reported no orthopedic or neurologic conditions that would impact testing. Participants were excluded if they were left-handed or had marked cognitive impairment on the Montreal Cognitive Assessment. In a single 45-minute session, quantitative cognitive and physical measures were collected in the Movement and Performance Sciences Laboratory at the Eugene Applebaum College of Pharmacy and Health Sciences, Wayne State University. All participants signed informed consent approved by the Wayne State University Institutional Review Board.

### Screening tools

The Edinburgh Handedness Inventory (EHI) is a 12 item self-report questionnaire in which the participants reported which hand is preferred for writing, drawing and other tasks. A score greater than 0.7 was used as a cut off point for participants to be considered right handed [18].

Montreal Cognitive Assessment (MoCA): Participants who scored below 26/30 were excluded from the study. The MoCA assesses multiple domains of cognition, has high test-retest reliability (correlation coefficient = 0.92), good internal consistency (Cronbach alpha = 0.83), and detects mild cognitive impairment with high specificity (87%), and sensitivity (100%) [19].

Completion of a health history questionnaire was followed by performance of the grip strength and cognitive assessments.

### Grip strength

Grip strength was quantified using a JAMAR hand dynamometer and standardized protocol [20]. Right and left maximum grip strengths were alternated across three trials with a one minute rest break between trials to prevent fatigue. The mean of three trials for each hand was used to determine overall grip strength. Grip strength on the non-dominant side was used to analyze correlations with cognitive measures.

### Cognitive assessments

Cognitive function was assessed using tests specifically related to cognitive decline in MCI and AD [2,6,7].

The Symbol Digit Modalities Test (SDMT) is a measure of processing speed. Participants were asked to match numbers to the correct geometric symbol by using a reference key. The number of correct matches performed in 90 seconds were recorded.

The Trail Making Test (TMT) tested attention, speed and mental flexibility. In TMT Part A, participants were required to connect 25 encircled numbers in a serial order as quickly as possible. In TMT Part B, participants were instructed to connect both encircled numbers and encircled letters in an alternating fashion (i.e., 1-A, 2-B, etc.) as quickly as possible. The length of time to complete TMT Part A and Part B was recorded.

The California Verbal Learning Test (CVLT) tested working memory by requiring participants to recall a list of 16 words. The list of words was read 5 times, and the participant repeated as many words as they could recall after each reading. Next, a second set of words was read to the participant and the participant repeated as many words as possible from this distractor list. Finally, after a 20-minute break, the participant was asked to list as many words from the first list as they could recall. The number of words recalled from the list was summed and z-scores were calculated using a custom software package.

The Controlled Oral Word Association Test (COWAT) tests semantic categorization and phonemic fluency. Participants were given a letter of the alphabet (C, F, L) and were instructed to list as many words as possible that begin with the stated letter in 60 seconds. The COWAT test produces several scores: the COWAT sum score (number of unique words produced across all three letters) and the COWAT total cluster score, in which clustering refers to a temporal mediated process that demonstrates an individual's ability produce words related to each other. The COWAT demonstrates moderate test-retest reliability r = 0.70 [21].

The Stroop Test tests executive function and response inhibition with three parts. In the Stroop Color test, participants were presented with a series of colored blocks printed on a sheet of paper and were asked to read aloud the color of each block. Next, the participant was presented with a sheet of paper with the names of colors written in black ink (e.g., RED) and instructed to read the names of the colors as quickly as possible (Stroop Word). Finally, the participant was presented with a sheet of paper on which the names of colors are written in incongruent ink (e.g., RED written in green ink) and instructed to name the color of the ink rather than read the word itself. The Stroop Interference score was defined as the number correct in 90 seconds.

### Power analysis

We a priori calculated the number of participants needed to achieve sufficient power using a G-Power analysis (Universität Düsseldorf, Düsseldorf, Germany). To determine effect size, we used a 2013 cross-sectional study examining relationships between grip strength and cognition in elderly men, similar to this study. Using the correlation coefficient for this relationship, the calculated effect size was 0.68 [13]. Using an alpha level of 0.05 and a power level of 95%, the analysis showed that the sample size needed to establish significance at this power level was 18 participants per group.

### Statistical analysis

All statistics were performed using SPSS version 25. Descriptive analyses were performed on all outcome measures. Independent t-tests were performed to determine between group differences and Pearson’s r correlations were used to analyze the relationship between the grip strength and cognitive measures. Further, Pearson’s r correlation was completed to examine how these relationships differed between the young and middle age groups.

## Results

Fifty-one individuals were recruited for this study. Five participants were ineligible to participate in the study as they did not meet the inclusion criteria for cognition (n = 4) or were colorblind (n = 1). Twenty-five young and 21 middle-aged, right-handed individuals with a baseline cognition score of 26/30 or greater on the MoCA participated in the study (Table 1).

**Table 1:** Participants.

	Young (n = 25)	Middle-aged (n = 21)
Age (years)	24.28 ± 1.7	54.1 ± 5.4
Sex (M:F)	15:10	4:17
EHI	.85 ± .07	.90 ±.09
MoCA (30 max)	27.3 ± 1.3	27.9 ± 1.3
Education (years)	17.88 ± 1.2	19.2 ± 4.3
Family History of cognitive impairment (%)	40%	42.9%

Mean ± SD for participant demographics. EHI- Edinburgh Handedness Inventory; MoCA—Montreal Cognitive Assessment

### Grip strength

For young individuals, no statistically significant differences were found between the right (47.7 ± 12.3 kg) and left (45.6 ± 10.4 kg) grip strength (p > .05). For middle-aged individuals, statistically significant differences were found between the right (40.1± 11.1kg) and left (36.1± 9.8 kg) grip strength (p < .01). Between group differences were also found for right (p < .05) and left (p < .01) grip strength. The middle-aged group showed lower right and left grip strength when compared to young individuals.

### Cognitive performance

The middle-aged group did significantly worse (14%) on the Stroop interference task when compared to young individuals (p < .05). No significant differences were found between the young and middle-aged group for the MoCA, EHI, COWAT, CVLT, SDMT, Trails A, and Trails B scores (Table 2).

**Table 2:** Grip strength and cognitive assessments in young and middle-aged adults.

Physical measure	Young (n = 25)	Middle-aged (n = 21)
Grip Right (kg)*	47.7 ± 12.3	40.1 ± 11.0
Grip Left (kg)**	45.6 ± 10.4	36.1 ± 9.8
**Cognitive measures**		
COWAT Total Cluster (#)	15.12 ± 6.4	20.52 ± 14.1
COWAT Adjusted (#)	42.7 ± 9.4	49.19 ± 13.1
Stroop Color (#)	83.72 ± 1.6	77.57 ± 11.1
Stroop Word (#)	101.36 ± 18.1	100.71± 21.4
Stroop Interference (#)*	48.96 ± 6.9	42.24 ± 9.9
Trails A (s)	22.23 ± 7.4	24.37 ± 7.2
Trails B (s)	43.28 ± 17.7	45.15 ± 14.1
SDMT Total (# correct)	66.0 ± 13.5	60.6 ± 8.0
CVLT Total (# words)	50.0 ± 8.7	49.6± 11.3
CVLT Long Delay (# words)	.10 ± .95	-.10 ± 1.0

Mean ± SD for grip strength and cognitive assessments in young and middle-aged adults. Significant differences were found between right and left grip strength in the middle-aged group (p < .01); Between group differences were found for the right (p < .05)* and left (p < .01)** grip strength and Stroop interference (p < .05)* COWAT Controlled Oral Word Association Test; SDMT -Symbol Digit Modalities Test; CVLT—California Verbal Learning Test

### Correlations between grip strength and cognitive performance

**Fig 1:**
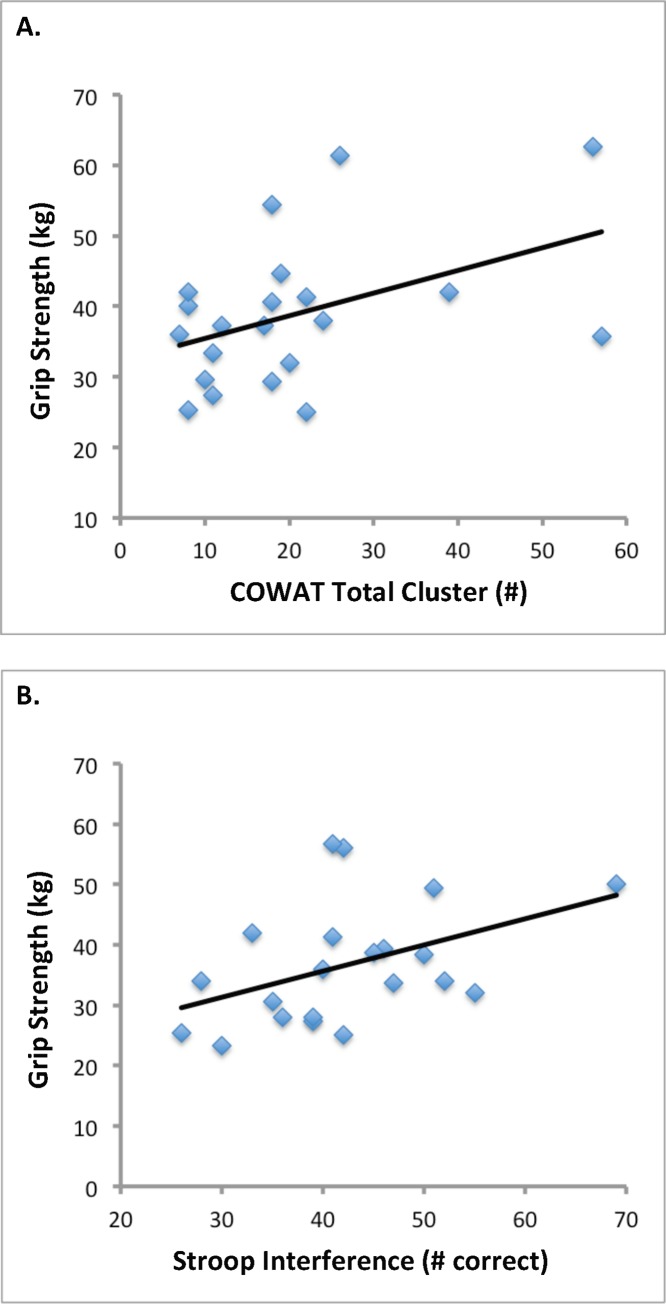
Relationships between grip strength and cognitive function in middle-aged adults. Middle-aged adults demonstrated a significant relationship between grip strength and A) COWAT total cluster score and B) Stroop Interference score.

For middle-aged individuals, grip strength was associated with the Stroop Interference (r = .471; p = .031 or p < .05) and COWAT scores (r = .458; p = .037 or p < .05) (Fig 1). Grip strength was not associated with other cognitive measures in middle-aged individuals. Further, no associations were found between cognitive measures and grip strength in the young individuals. (Table 3)

**Table 3:** Correlations between grip strength and cognitive measures in young and middle-aged adults.

	Young	Middle
	Grip strength	
COWAT Total Cluster	r = .06	r = .458*
Stroop Interference	r = -.207	r = .471*
Trails A	r = .059	r = .08
Trails B	r = .190	r = -.06
SDMT Total	r = .09	r = .16
CVLT Total	r = .124	r = .17
CVLT Long Delay	r = .09	r = .25

Correlations between grip strength and cognitive measures in young and middle-aged adults. Significant correlations were found for grip strength and COWAT total cluster and Stroop interference in the middle-aged group (p < .05)* COWAT Controlled Oral Word Association Test; SDMT -Symbol Digit Modalities Test; CVLT—California Verbal Learning Test

## Discussion

The purpose of this exploratory study was to investigate the relationship between grip strength and cognitive function in young and middle-aged adults. Although previous studies have examined the relationship between grip strength and global cognition for participants 65 years or older, [2, 3, 9, 11–14] very few studies [22, 23] have examined such relationships in younger and middle-aged adults or examined specific domains of cognition. Our results indicate that middle-aged adults demonstrate significantly weaker grip strength compared to young adults. This finding aligns with published norms for grip strength indicating that grip strength peaks at age 30–40 and then declines with increasing age [24,25]. Reduced grip strength in middle age has also been explained by the association of risks factors such as stress or having a chronic disorder [11] though these measures were not evaluated in the present study. Our results indicate that middle-aged adults demonstrate significantly poorer performance on the Stroop Interference test compared to young adults. The Stroop Interference score has been shown to decrease with age, representing delayed cognitive abilities and executive function with age [26].

We examined relationships among specific cognitive domains and grip strength. In the younger-aged group, no relationships were found, while in middle-aged adults there were significant relationships among grip strength and poorer performance on both Stroop Interference and COWAT total cluster score. No correlations were found between grip strength and the CVLT, SDMT, or TMT in middle-aged adults. This finding builds on prior literature in both older adults [4] and middle-aged adults [23] demonstrating that executive function, but not other cognitive domains, was associated with steeper decline in gait speed, another functional marker. Interestingly, executive dysfunction has been found earlier in the disease course of MCI and AD [27, 28] compared to other cognitive domains. Executive dysfunction has also been found as a contributor to functional impairment [28]. Our finding of a positive correlation between grip strength and Stroop Interference scores of middle-aged adults suggest that declines in grip strength are correlated with declines in executive function. This may suggest that declines in executive function can be monitored earlier in the aging process by using grip strength as a biomarker in order to better identify participants at risk for cognitive decline. Phonemic fluency, along with executive function, has been found to be an effective neuropsychological measure for differentiating between mildly impaired AD participants and normal controls [29]. Phonemic fluency or semantic categorizations was assessed with the COWAT total and COWAT Total Cluster score, which has been found to be correlated with grip strength scores in other studies of older adults [4]. In particular, Stroop Interference and COWAT Total Cluster may indicate higher level processing required for response inhibition and categorizing words by clusters. As such, these domains may be highly sensitive to early changes in cognition, though future larger scale longitudinal studies are needed to confirm this hypothesis.

The CVLT and the TMT assess working memory and attention respectively. A study investigating the attentional control of working memory in AD and MCI found that AD participants had significant impairment on all three attentional tests measured, while MCI participants had impairments on only one attentional test [27]. Their results suggest individuals with MCI experience a decline in attentional control processes of working memory. Since our middle-aged participants did not have any correlations between grip strength and working memory and attention, this may suggest that declines in these domains do not contribute to functional impairments. Also, differential effects on cognitive domains due to cognitive heterogeneity that is present in the early phases of AD and MCI may contribute to differences in performance of cognitive tests and presentation of cognitive subtypes [27].

This study was limited by both a small sample size and a convenience sample. Further, our sample was highly educated; several middle-aged participants had greater than 22 years of education, which may have resulted in a ceiling effect of the COWAT. However, the educational level of our sample is similar to other studies [30–31]. Further, there is conflicting evidence with regard to rate of cognitive decline and educational level. Although some studies have found that higher education was related to slower cognitive decline [32–33], this idea has been recently challenged by studies showing no associations between education, cognitive decline and cognitive reserve [34–36].

In conclusion, this study showed that specific domains of cognition may be more sensitive to detection of early cognitive declines than global measures and that grip strength may serve as biomarker for detecting for early cognitive changes in middle-aged adults. The findings of this study further justify the inclusion of grip-based measures in future randomly selected, nationally representative longitudinal designs to establish a predictive relationship between cognitive performance and grip strength of middle-aged adults.

## Supporting information

S1 File De-identified dataset. (XLSX)
